# Leptin Resistance and Cardiometabolic Disorders: Bridging Molecular Pathways, Genetic Variants, and Therapeutic Innovation

**DOI:** 10.2174/011573403X356019250118170444

**Published:** 2025-02-07

**Authors:** Prashanjit Roy, Rishi Kant, Amandeep Kaur, Hardik Kumar, Ranjeet Kumar

**Affiliations:** 1 Department of Pharmacy Practice, ISF College of Pharmacy, Moga, Punjab, India;; 2 Department of Pharmacy Practice, Narayan Institute of Pharmacy, Gopal Narayan Singh University, Sasaram-Tilauthu Rd, Rohtas, Bihar, 821305, India

**Keywords:** Leptin, CVD, hypertension, obesity, type 2 diabetes, hypothalamus

## Abstract

Leptin, a hormone produced by fat cells, is crucial for regulating energy equilibrium, managing body mass, and influencing metabolic and cardiovascular well-being. Leptin decreases appetite, boosts energy usage, and has a significant impact on glucose metabolism by primarily activating the JAK2/STAT3 signaling pathway in the hypothalamus. Obesity leads to the development of leptin resistance, which is marked by high levels of leptin in the bloodstream and a decreased responsiveness to its signals. This leads to increased food consumption, weight gain, and metabolic issues, such as type 2 diabetes (T2DM) and cardiovascular disease (CVD). This study explores the many roles of leptin in metabolic regulation, with a specific emphasis on its interaction with insulin and its impact on peripheral organs like the pancreas, liver, and muscles. Leptin resistance worsens chronic inflammation, oxidative stress, endothelial dysfunction, and insulin resistance, all of which are strongly linked to the development of cardiovascular disease (CVD). Moreover, there is a correlation between genetic variations in the leptin receptor (LEPR) gene and a higher susceptibility to stroke and other cardiovascular issues. Therapeutic interventions, such as leptin replacement therapy, have demonstrated potential in the treatment of congenital leptin insufficiency and lipodystrophy while also enhancing glycaemic control, lipid profiles, and neuroendocrine function. Recent studies have indicated that manipulating leptin levels or enhancing its responsiveness by specific treatments, such as chemical chaperones and inhibitors of negative regulators like SOCS3 and PTP1B, might potentially restore the efficacy of leptin.

## 1 INTRODUCTION

Leptin, a hormone that is crucial for maintaining energy balance and body weight, works by influencing blood pressure, heart rate, and vascular function, thereby having a substantial impact on cardiovascular health [1, 2]. Increased cardiac inflammation, fibrosis, hypertension, and disrupted cardiac metabolism are all associated with leptin resistance, which is frequently observed in obesity [3]. This resistance results in excess weight gain. Rodents with leptin mutations and animals with lipodystrophy have demonstrated that the treatment of leptin deficiencies can result in significant weight loss [4, 5]. The Janus tyrosine kinase 2 (JAK2)/cytosolic signal transducer and activator of transcription protein 3 (STAT3) pathway is the most well-characterized pathway mediated by leptin receptors in the hypothalamus [6]. Excess circulating levels of leptin and decreased responsiveness to the hormone are characterized by leptin resistance, which is frequently linked to an increase in fat deposits and a higher body weight (BMI) [7]. BMI and leptin levels are significantly correlated, with higher BMI typically corresponding to greater concentrations of leptin [6]. Understanding these mechanisms could lead to new therapeutic strategies for managing diabetes and other metabolic disorders [8]. High leptin levels did not substantially increase the risk of CVD or stroke once other risk factors for cardiovascular disease (CVD) were taken into consideration, according to research on the correlation between the two [9]. However, the findings were mixed, and certain variations in the leptin receptor (LEPR) gene were associated with a higher risk of CVD, particularly with stroke [10]. An elevated risk of cardiovascular disease is associated with resistance to insulin, chronic inflammation, and leptin resistance, which is frequently observed in obesity [11]. High levels of leptin are associated with impaired arterial distensibility and proinflammatory cytokines TNF-Î± and IL-6, which are linked with insulin resistance and endothelial dysfunction, potentiating CVD [12, 13]. Certain polymorphisms in genes that code for the leptin receptor are strongly associated with the risk of CVD, particularly stroke [14]. Leptin is a key hormone in the body, and its levels are positively related to hypertension in both sexes, regardless of body weight [15]. The leptin signaling networks, which involve the JAK2/STAT3 pathway, have a vital function in controlling the equilibrium of energy, glucose metabolism, immunological responses, and several physiological processes [16, 17]. Leptin resistance, which hinders the phosphorylation procedure of JAK2, an essential process within the leptin signaling pathway, is associated with obesity [18]. SOCS3 is crucial in facilitating leptin resistance by obstructing leptin signaling pathways, and higher levels of SOCS3 expression are associated with leptin resistance in obesity models. In obesity, chronic high leptin levels can lead to leptin resistance, where the hypothalamus becomes less responsive to the regulatory effects of leptin, resulting in disrupted appetite control and energy imbalance. This resistance is often driven by the overexpression of SOCS3 (suppressor of cytokine signaling 3), which further impairs leptin signaling and contributes to elevated blood pressure by activating the sympathetic nervous system (SNS). Leptin resistance is associated with increased inflammation and a higher risk of cardiovascular disease (CVD) and stroke, partly due to the involvement of PTP1B (protein tyrosine phosphatase 1B), a negative regulator of leptin signaling. As leptin resistance progresses, it disrupts key metabolic functions, leading to a cascade of consequences that drive obesity and diabetes. The brain receives weaker satiety signals due to impaired leptin signaling, causing increased appetite and overeating, which adds to body fat stores. At the same time, reduced energy expenditure occurs, as the body stores more energy as fat instead of burning it. This leptin resistance also interferes with normal glucose metabolism, leading to dysregulated glucose production and elevated blood glucose levels, a condition known as chronic hyperglycemia. Additionally, leptin resistance lowers fatty acid oxidation, meaning that the body breaks down fewer fatty acids, further increasing fat accumulation. These combined effects trigger oxidative stress due to a buildup of reactive oxygen species (ROS) and cause chronic inflammation, both of which harm cells and tissues over time. Importantly, these stressors impair pancreatic beta cells, which produce insulin, further diminishing the body’s ability to regulate blood glucose. This worsening glucose control and metabolic imbalance contribute to the development of type 2 diabetes, emphasizing the intricate links between leptin resistance, obesity, and metabolic dysfunction, as shown in Fig. (**1**) [19, 20]. Phosphoinositide 3-kinase (PI3K) is another essential enzyme influenced by leptin and other androgens, and its activation in the hypothalamus can elicit both activating and inhibitory responses on PKC [21]. Reducing SOCS3 activity could be a potential therapeutic strategy for improving leptin sensitivity and managing obesity [5, 22]. Leptin replacement treatment has shown promise in treating metabolic and endocrine illnesses, mainly those marked by a lack of leptin, for instance, lipodystrophy and congenital leptin deficiency [23]. It improves glycaemic control, lipid profiles, and neuroendocrine function [24]. However, challenges persist in overcoming leptin resistance, particularly in common obesity [25]. Decreasing circulating leptin levels can enhance leptin sensitivity and various metabolic processes, as evidenced by studies on CRISPR-Cas9-loxP and leptin-neutralizing in mouse models [26]. Treatments aimed at alleviating endoplasmic reticulum stress, including specific chemical chaperones like 4-phenyl butyric acid and tauro-ursodeoxycholic acid, can function as sensitizers to leptin and enhance its signaling pathways [22]. Melatonin, known for its properties in counteracting oxidative stress and inflammation, has emerged as a promising approach for addressing obesity linked to leptin resistance [27]. Targeting specific phosphatases that adversely influence leptin signaling and managing hypothalamic inflammation can contribute to the restoration of leptin sensitivity, underscoring the complex nature of leptin resistance and identifying potential therapeutic strategies [28, 29]. This review examines the intricate involvement of leptin in obesity, cardiovascular disease, and type 2 diabetes, emphasizing its impact on metabolic and cardiovascular well-being [30]. It also discusses the possible therapeutic advantages of targeting leptin signaling pathways [31]. Leptin was first identified in 1994 by scientists Jeff Friedman, Douglas Coleman, and Andrew Myers at Rockefeller University during their investigation of the ob/ob (obese) mouse [32, 33]. Recent research has highlighted the potential role of leptin in cardiovascular disorders, emphasizing its significance in this area of study [34]. Leptin binds to LEPR, affecting appetite and energy. This review examines these elements, making it an essential field of research for understanding the relationship underlying metabolic processes and cardiovascular health [20]. It explores the intricate involvement of leptin in overweight and obesity, heart attacks, and type 2 diabetes, emphasizing its impact on both metabolic and cardiovascular well-being [35].

## 2 THE RELATIONSHIP BETWEEN LEPTIN AND OBESITY

Pro-opiomelanocortin (POMC) and Agouti-related peptide (AgRP) neurons are very important in the regulation of glucose homeostasis and the maintenance of energy balance [36]. POMC neurons, which are found in the nucleus arcuate of the hypothalamus, are responsible for blocking appetite and increasing energy expenditure. Leptin inhibits hunger in the hypothalamus and other regions of the brain when energy is abundant. Leptin and gastrointestinal hormones, such as CCK, enhance satiety and reduce food consumption, hence promoting long-term control of eating habits and maintaining energy equilibrium, as shown in Fig. (**2**) [37]. They do this by producing melanocortin, which are hormones that suppress hunger [38]. Alternatively, AgRP nerve cells, which are similarly located in the arcuate nucleus, are responsible for stimulating hunger and reducing energy expenditure *via* the process of inhibiting melanocortin receptors [39]. On the other hand, insulin, which is released by the pancreas and leptin, which is generated by adipose tissue, both affect these neurons to control food intake and energy balance [40]. To preserve the energy equilibrium and glucose levels of the body, a connection between the two neurons and chemicals, such as leptin, along with insulin, is very necessary [41].

## 3 ENERGY-BALANCING LEPTIN MECHANISMS

Administering treatment for leptin deficits, whether complete or partial, may result in a significant reduction in body weight. This has been seen in mice with leptin alterations and animals afflicted with lipodystrophy [17]. In addition, a genetic alteration associated with fat-specific long non-coding RNA, LncOb, leads to an augmentation in adipose tissue and a decrease in leptin concentrations [42]. Furthermore, the administration of leptin to these mice results in significant weight reduction [43]. The JAK2/STAT3 pathway is the most well-characterized system regulated by leptin receptors in the hypothalamus and is the most extensively studied system involving the Janus tyrosine kinase 2 (JAK2) and cytoplasmic signal transduction and activation of transcriptional protein 3 (STAT3) [44]. The signaling process in the hypothalamus, regulated by the leptin receptor, involves many routes. Among them, the JAK2/cytosolic STAT3 pathway is the most well-studied and understood [45]. Multiple pathways play a role in hypothalamic signaling regulated by the leptin receptor, but the cytosolic activator and signaling transducer of transcription protein 3 (STAT3) are the most extensively studied [46].

## 4 THE SIGNALLING MECHANISM OF LEPTIN RECEPTOR

The regulation of this process is carefully controlled by systems that provide feedback that is positive as well as negative. The JAK2/STAT3 pathway is crucial for the signaling mechanism of leptin, which binds to its receptor (LepR) on the surface of hypothalamic neurons. Upon binding, leptin activates JAK2, which, in turn, phosphorylates STAT3. Once phosphorylated, STAT3 dimerizes and translocates to the nucleus, where it regulates the expression of genes involved in energy homeostasis and appetite control. This tightly regulated process is essential for maintaining energy balance and is modulated by a network of feedback mechanisms [47]. By interfering with the JAK2/STAT3 pathway, negative regulators like SOCS3 and PTP1B can suppress leptin signaling. This pathway is tightly regulated by feedback mechanisms to maintain balance. Negative regulators, such as SOCS3 and PTP1B, act as molecular brakes on leptin signaling. SOCS3 binds to JAK2, preventing further phosphorylation, while PTP1B dephosphorylates JAK2 and STAT3, thereby reducing their activity. Together, these regulators inhibit leptin signaling, which can decrease leptin sensitivity and contribute to conditions like obesity and metabolic dysfunction [48]. On the other hand, the positive regulator Src-homology 2 domain 1 (SH2B1) boosts the activity of JAK2, which, in turn, increases the amount of leptin signaling that occurs downstream of JAK2. In addition, positive regulators like SH2B1 enhance the activity of JAK2, ensuring efficient leptin signaling. SH2B1 binds to JAK2, promoting its phosphorylation and activation, which subsequently increases the levels of phosphorylated STAT3. This leads to enhanced transcription of genes related to energy balance and appetite control. By supporting proper leptin signaling, SH2B1 helps maintain energy equilibrium and overall metabolic health [49]. However, stress in the endoplasmic reticulum (ER) may interfere with the JAK2/STAT3 pathway, which, in turn, reduces leptin sensitivity and contributes to obesity *via* its interaction with the PTP1B [50].

## 5 THE CONNECTION BETWEEN OBESITY AND LEPTIN RESISTANCE

Leptin, a key regulator of appetite and energy balance through leptin receptors (LEPR) in the hypothalamus to suppress hunger and promote energy expenditure, becomes disrupted in obesity, leading to impaired leptin signaling [51]. As fat stores increase, leptin levels rise, resulting in a state known as hyperleptinemia. Despite the elevated leptin, the hypothalamus becomes less responsive to its signal, leading to leptin resistance. This resistance occurs due to the desensitization of LEPR, caused by prolonged exposure to high leptin levels, which diminishes the receptors' ability to regulate appetite and energy expenditure effectively. As a result, the body’s mechanisms for balancing food intake and energy use are compromised, contributing to the cycle of overeating and reduced energy expenditure that characterizes obesity [52]. Insulin, produced by the pancreas, signals satiety *via* INSR (insulin receptors) in the hypothalamus, activating the PI3K/Akt pathway and mTOR to regulate energy expenditure, but due to insulin resistance, the hypothalamus's ability to control hunger and energy use is reduced, promoting overeating. Ghrelin, the hunger hormone, activates GHSR-1a receptors in the hypothalamus, stimulating the MAPK pathway and increasing the release of NPY and AgRP, which promote food intake. However, the dysregulated ghrelin secretion, with elevated levels at inappropriate times, overrides leptin and insulin signals, reinforcing weight gain. Adiponectin, which enhances insulin sensitivity and energy expenditure *via* AMPK (AMP-activated protein kinase), is reduced in obesity, impairing fat oxidation and exacerbating weight gain. Even ER stress in hypothalamic neurons disrupts leptin and insulin signaling, with the UPR pathway involving IRE1, PERK, and ATF6 triggering inflammation that impairs receptor function. This leads to leptin resistance and insulin resistance, perpetuating obesity by impairing the brain’s regulation of appetite and energy balance [53, 54]. Genetic factors also significantly contribute to leptin resistance, influencing both receptor function and overall energy regulation. Disruptions in SLC32A1 can alter neurotransmitter signaling, impairing leptin’s effectiveness in controlling eating behaviors and energy expenditure [55]. GCKR plays a significant role in the metabolism of carbohydrates and the production of insulin, both of which have the potential to influence glucose levels. GCKR can indirectly influence how the body utilizes lipids and controls leptin levels through its effects on energy balance. With variations in GCKR potentially altering how the body stores and utilizes energy, it may subsequently lead to metabolic diseases and obesity [56]. A significant genetic factor contributing to obesity is the FTO (fat mass and obesity-associated) gene, which encodes an enzyme that regulates RNA demethylation. The FTO enzyme is involved in the regulation of mRNA stability and expression of genes related to metabolism and fat storage. Variants of the FTO gene increase the likelihood of overeating, particularly high-calorie foods, and reduce energy expenditure, contributing to weight gain. The FTO enzyme impacts the central nervous system by influencing areas of the brain that control appetite, and its variants are linked to obesity susceptibility [57]. The other known gene related to obesity is IRX3 (Iroquois Homeobox 3), which is located near FTO and is involved in regulating fat storage and distribution. IRX3 affects how fat is distributed in the body, particularly in the visceral fat region, which is closely linked to metabolic diseases. Changes in IRX3 function can alter the body’s response to energy regulation and lead to an increased likelihood of fat accumulation [58]. Inflammation and endoplasmic reticulum (ER) stress play critical roles in the obesity mechanism. Pro-inflammatory cytokines (*e.g*., TNF-α and IL-6) are released by adipose tissue in obesity and interfere with leptin signaling pathways, reducing the ability of the brain to respond to leptin’s signals to suppress hunger and increase energy expenditure. These mechanisms form a complex feedback loop, where leptin resistance leads to further weight gain, reinforcing the cycle of obesity. As leptin levels rise, the hormone travels to the brain, where it binds to leptin receptors (ObRb) in specific areas of the hypothalamus. This binding triggers the JAK2/STAT3 signaling pathway, a critical step in the regulatory role of leptin. Within this pathway, JAK2 and STAT3 are phosphorylated, allowing STAT3 to move into the nucleus and regulate gene expression. The resulting gene activity leads to decreased food intake, lower body weight, and an increase in energy expenditure, promoting energy balance. A feedback loop involving the protein SOCS3 helps to prevent overactivation of leptin signaling by inhibiting further stimulation, maintaining a controlled balance and ensuring leptin sensitivity in a lean individual. In the obese phenotype, it is characterized by leptin resistance, a condition where leptin signaling is impaired despite high levels of leptin (leptinemia) in the bloodstream. The excess leptin struggles to access the central nervous system (CNS) due to physical or signaling barriers, limiting its effects. Within the brain, specifically at the level of the leptin receptor (ObRb), downstream signaling becomes impaired and ObRb expression on cell membranes decreases, and negative regulatory factors like SOCS3 are upregulated, further hindering the signaling pathway. Additionally, hypothalamic inflammation and endoplasmic reticulum (ER) stress exacerbate this impairment by disrupting the effects of leptin on neurons. These factors collectively lead to leptin resistance, where the hormone's ability to regulate body fat (lipostatic action) is lost. Consequently, the body fails to curb food intake or manage energy balance effectively, contributing to continued weight gain and energy imbalance in obese individuals, as shown in Fig. (**3**) [59].

## 6 THE ROLE OF LEPTIN IN DIABETES

Due to its influence on the volume of food consumed and weight, leptin is also accountable for regulating the quantity of glucose in the body, which plays a crucial role [60]. To regulate glucose metabolism, it imposes the impact it has not only on the brain but also on peripheral organs, such as the pancreas, liver, and muscles. There is a correlation between leptin and the release of insulin through pancreatic beta cells, which is a critical process for maintaining glucose homeostasis [61]. Additionally, it helps reduce blood sugar levels by decreasing the synthesis of glucagon and corticosterone, accelerating the amount of glucose that is taken in by the body, and blocking the release of glucose generated by the liver [62]. The influence of leptin on glucose control includes several different signaling pathways, including those that are reliant on STAT3 as well as others that are independent of it. The canonical Wnt pathway is the method by which leptin regulates glucose levels in zebrafish, providing a novel mode of action [63]. By highlighting the significance of central leptin signaling, it is important to note that the hypothalamic arcuate nucleus is an essential location for the influence that leptin has on glucose homeostasis. A substantial portion of the anti-diabetic effects of leptin is played by the central nervous system leptin-melanocortin system, which is notably involved in the proopiomelanocortin neurons and melanocortin-4 receptors [64]. In general, leptin plays a significant role in the control of insulin secretion, the suppression of counter-regulatory hormones, and the maintenance of glucose homeostasis. It does this by acting in the brain and peripheral tissues [65]. Important parts of the brain, such as the hypothalamic arcuate nucleus, are involved in the regulation of its effects, which are mediated by several different signaling pathways, including the STAT3 and Wnt pathways [66]. Having a better understanding of these pathways may result in the development of novel treatment approaches for the management of metabolic illnesses, such as diabetes. These processes may lead to new treatment methods for managing metabolic illnesses, such as diabetes [67].

## 7 EFFECTS ON INSULIN SENSITIVITY AND SECRETION

Leptin imposes a substantial contribution and immediate inhibitory effect that impacts the release of insulin, presumably triggering disruptions in carbohydrate metabolism within adipose tissues that are excessive in fat [68]. Cdc42 has a significant role in age-related obesity and could be specifically addressed using antidiabetic medicines or antioxidants to aid in weight reduction among overweight or obese persons [69]. The leptin receptor is present in the pancreatic islets and might potentially affect both the basal and glucose-stimulated release of insulin when it is generated in large quantities by abdominal fat tissue. Leptin not only regulates fat but also has an impact on hemopoiesis and macrophage activity [70]. The compounds UK-14,304 and noradrenaline have been found to significantly decrease the secretion of insulin from transplanted pancreatic islets. This suggests that there is a modification in the functioning of alpha-2 adrenoceptors on beta-cells [71]. Leptin can impact the functioning of insulin in individuals who are obese by reducing the activities triggered by insulin and raising the activity of phosphatidylinositol 3-kinase associated with IRS-1 [72]. Lean individuals with a low amount of adipose tissue have a higher concentration of bound leptin, which may diminish its influence on food consumption [73, 74]. Conversely, obese persons have a greater amount of free leptin, which may alter their resistance to its effects. Leptin receptors are found on pancreatic beta-cells, indicating that their binding could control insulin expression in a negative feedback loop, potentially assisting in weight loss [75]. There is a connection between insulin resistance and increased levels of leptin in the blood, regardless of the amount of body fat. However,the creation of leptin is not directly affected by insulin in the blood [76]. Leptin receptor mutations can cause diabetes and indicate that three STAT proteins may be involved in mediating the anti-obesity benefits of leptin.

## 8 CLINICAL RESEARCH

The fat-cell-produced hormone leptin articulates promise as a possible medication for those suffering from type 1 and type 2 diabetes. Its ability to improve blood sugar regulation is independent of both weight loss and calorie intake [77]. In addition to lowering the high glucagon levels linked with diabetes, leptin prevents the buildup of harmful fat in organs. Scientific investigations have linked leptin levels to an increased risk of developing type 2 diabetes, suggesting that insulin resistance plays a major role in this condition. Treatment of obese diabetic rats with leptin improved insulin sensitivity and allowed for better control of blood sugar levels, independent of the animals' dietary habits [78]. Curiously, whereas higher leptin levels are associated with a higher risk of type 2 diabetes in men, no such connection is seen in women. In addition, there is evidence linking a specific variant in the LEPR gene to an elevated risk of acquiring type 2 diabetes. This research emphasizes the need to offer personalized medical care to those with this genetic mutation to prevent the emergence of diabetes [79].

## 9 THE RELATIONSHIP BETWEEN LEPTIN AND CVD

The hormone leptin has been linked to several metabolic and cardiovascular disorders, and it is essential for maintaining a healthy energy balance. The correlation between leptin and CVD has been the subject of contradictory studies. After other cardiovascular risk factors were taken into consideration, it was found that high levels of leptin did not substantially raise the risk of coronary heart disease (CHD) or stroke [80]. Several variants in the LEPR gene were linked to an increased risk of cardiovascular disease, including stroke. Another possible way that leptin might trigger cardiovascular disease and the progression of atherosclerosis is that it is associated with decreased arterial flexibility when levels of the hormone are high [81]. Damage to metabolic and inflammatory organs, including the cardiovascular system, may result from resistance to insulin, persistent inflammation, and a higher probability of cardiovascular disease (CVD), all of which are associated with leptin resistance, which is prevalent in obese people [82]. Animal research has shown that leptin raises blood pressure by increasing sympathetic nerve activity. However, human studies on the same subject have revealed conflicting results. Although the connection was not statistically significant after controlling for body mass index (BMI), higher leptin levels were linked to an increased risk of cardiovascular disease and CVD events in older persons [83]. In addition, there is evidence that leptin levels are associated with coronary heart disease alongside various non-cardiac vascular conditions and that people with abdominal obesity are at a higher risk of cardiovascular disease due to an imbalance between leptin and adiponectin levels [84].

## 10 THE CORRELATION BETWEEN ARTERIAL DISEASE AND HIGH BLOOD PRESSURE ASSOCIATED WITH LEPTIN

Even after controlling for other adipokines, body mass index, and other risk factors, an increase in leptin is linked with a rise in blood pressure and an increased likelihood of hypertension. Although it is much greater in males, it is unaffected by factors, such as race, ethnicity, or smoking status [85]. Thus, elevated leptin levels may be associated with a pro-oxidant state and decreased nitric oxide levels, as is typical in hypertension caused by obesity. Atherosclerosis, stroke, coronary artery disease, and peripheral artery disease are all linked to increased levels of circulating leptin, which, in turn, increases the risk of these cardiovascular events [86]. In reality, leptin promotes dysfunctional endothelial cells, inflammatory conditions, oxidative stress, and cardiovascular smooth muscle cell proliferation, all of which contribute to atherogenic consequences. Elevated leptin levels are linked to inflammatory biomarkers and subclinical atherosclerosis in SLE patients, suggesting an increased likelihood of cardiovascular problems [87]. Activation of the sympathetic nervous system, chronic oxidative stress, nitric oxide insufficiency, and accelerated renal salt reabsorption are some of the ways in which leptin affects blood pressure control [88]. Persistently elevated blood pressure results from impaired vasorelaxation and natriuresis, which is caused by chronic hyperleptinemia. The release of cytokines, including IL-6, TNF-α, and IL-17, is induced by leptin, which enhances inflammation and contributes to the development of atherosclerosis [89].

## 11 APPROACHES CONNECTING LEPTIN WITH CARDIOVASCULAR DISEASE

The association between high leptin levels and decreased vascular pliability has been established. On the other hand, excessive levels of leptin in the blood may be responsible for adverse effects on the vasculature as well as atherogenesis. In addition, leptin increases the levels of cytokines that promote inflammation, namely TNF-α and IL-6 [90]. These cytokines are linked to resistance to insulin and endothelial dysfunction, which further exacerbates cardiovascular disease. An ailment that is often referred to as leptin resistance is typically characterized by high levels of leptin in the blood and diminished leptin signaling signals [91]. Chronic inflammation, metabolic issues, and cardiovascular disease (CVD) are all effects that result from this. Given that some polymorphisms within genes that encode for the leptin transmitter are strongly associated with the risk of cardiovascular disease, particularly stroke, it would seem that there is a hereditary component that might potentially influence the effect that leptin activity has on the health of the heart [92]. This ratio has emerged as a critical biomarker of metabolic syndrome as a result of the proinflammatory state, along with a higher risk of cardiovascular disease that is generated by a low adiponectin level and an elevated leptin level [93]. On the other hand, high leptin levels have been associated with key indicators of risk for cardiovascular disease as well as fundamental components of metabolic syndrome, such as obesity and insulin resistance. In women of middle age, researchers have shown a link between the levels of leptin and the presence of molecular markers that are associated with atherosclerosis [94]. Furthermore, in a number of investigations, it has been shown that increased levels of leptin provide protection against mortality caused by cardiovascular disease. Any rise in leptin level in FCH patients, independent of their weight, body mass index, or insulin resistance, is associated with an increased risk of developing cardiovascular disease [95].

## 12 THE RELATIONSHIP BETWEEN LEPTIN AND HYPERTENSION

Through the blunting of the acute depressor impacts of other pressure processes, chronic hyperleptinemia contributes to the development of hypertension [96]. These mechanisms include increased oxidative stress, a deficit in NO, improved renal salt reabsorption, and an overproduction of endothelin. It is the sympathetic nerve activity that is stimulated by leptin, which results in general vasoconstriction and boosts kidney-specific effects on salt retention, particularly by the kidneys, the heart, and the adrenal glands [97]. Additionally, leptin is responsible for the elevation of both systemic and intrarenal oxidative stress, the diminution of bioactive nitric oxide (NO) *via* the process of breakdown produced by reactive oxygen species, and the maintenance of renal sodium retention through the stimulation of tubular sodium reabsorption [98]. Stimulation *via* the transient receptor potential melastatin 7 channels in the carotid body is increased by leptin, and the prevention of leptin-induced hypertension may be achieved by the inhibition of Trpm7 or through denervation of the carotid region [99]. In this manner, it increases the activity of the renin-angiotensin system and proinflammatory cytokines in the brain, so making the organism more susceptible to the hypertension that is caused by angiotensin II, especially when the diet is heavy in fat. Plasma leptin levels are positively related to arterial hypertension in both sexes, regardless of body adiposity [100]. However, certain genetic polymorphisms within the gene that codes for leptin and its receptor confer a genetically determined higher danger of developing hypertension. As a result, these polymorphisms provide an inheritable susceptibility to leptin-induced hypertension [101]. Additionally, this association involving leptin and hypertension is much greater in females, especially after menopause. This further suggests that there may be certain processes that are unique to women that are responsible for the function that leptin plays in controlling blood pressure. Sympathetic activation, oxidative stress, reduced bioavailability of nitric oxide, and renal salt retention are the components that make up the pathophysiology of hypertension that is produced by leptin [102]. The association between greater leptin levels and increased blood pressure, which is independent of body weight, is the data that supports this claim. This correlation exists at the clinical and genetic levels. Second, these variations between the sexes imply that the hypertensive effects of leptin may be more substantial in females than in men [103].

## 13 SIGNALING PATHWAYS INVOLVING LEPTIN

Under the influence of the JAK2/STAT3 pathway, leptin stimulates angiogenesis in RF/6A cells when they are cultured *in vitro*. There is a possibility that dysfunctions in the regulation of the PI3K-PDE3B-cAMP pathway might result in central leptin resistance in obese individuals [104]. This resistance plays a role in the genesis of obesity that is connected with diet and age. JAK/STAT signaling, which is associated with the mitochondrial permeability transition pore (MPTP), is responsible for the cardioprotective effects of leptin [105]. This signaling takes place sooner during reperfusion in comparison to Akt phosphorylation. Furthermore, leptin is able to increase the transcription of placental genes and cell proliferation without the need for a JAK-STAT pathway that is operating properly. Leptin signaling is necessary for the control of energy balance, as well as glucose and metabolism, immunological responses, and a variety of other body activities [106]. Additionally, it activates tyrosine kinases that are not members of the JAK2 family, such as members of the Src family, in order to phosphorylate JAK2, STAT3, and other molecules that are downstream of LEPRb [107]. Additionally, due to the stimulation of the JAK2/STAT3 signaling pathway, leptin causes human B cells to produce cytokines, which, in turn, enhances the inflammatory and immunoregulatory activities of these cells [108], as mentioned in Table **1**.

## 14 THE FUNCTION OF SOCS3 IN RESISTANCE TO LEPTIN

A key stage in the leptin signaling pathway is the phosphorylation of JAK2, which SOCS3 blocks, acting as a negative regulator of leptin signaling. A feedback loop inhibiting additional leptin signaling is established when leptin promotes SOCS3 expression [109]. In diet-induced obesity models, an increase in SOCS3 expression in the hypothalamus is associated with leptin resistance. Specifically, overexpression in neurons, such as POMC neurons, leads to obesity and leptin resistance. To lose weight and avoid diet-induced obesity, mice with brain cell-specific SOCS3 deletion showed increased leptin sensitivity [110]. Although it does not prevent obesity, a lack of SOCS3 in cells that express the leptin receptor makes them more sensitive to leptin and protects them against diet-induced insulin resistance. Improving leptin sensitivity and managing obesity may be possible by reducing SOCS3 activity [111]. Different kinds of neurons may influence how SOCS3 affects energy balance and leptin resistance; for instance, increasing its expression in POMC neurons leads to obesity and leptin resistance, whereas increasing its expression in leptin receptor neurons has no such effect [112]. Thus, SOCS3 is an important target for obesity management and leptin sensitivity enhancement because it inhibits leptin signaling pathways, which, in turn, mediates leptin resistance. Elevated SOCS3 expression is associated with obesity-related leptin resistance, and SOCS3 deficiency protects against diet-induced metabolic problems [113].

## 15 LEPTIN-ASSOCIATED PI3K

Phosphoinositide 3-kinase, often known as PI3K, is an enzyme that plays a crucial role in the regulation of several signaling pathways. It is regulated by insulin and leptin and serves as a point of junction for these networks [114]. The products of PI3K are responsible for activating protein kinases, including Akt and several different isoforms of PKC. When insulin attaches to its receptor, it attracts insulin receptor substrates (IRSs), which are then phosphorylated on tyrosine residues by the intrinsic kinase activity of the receptor. This ensures the production of insulin [115]. As a result of this phosphorylation, their ability to attach to other signaling molecules is increased, which, in turn, triggers the following events along the route. IRS2 proteins interact with the regulatory subunit p85 of PI3K, which results in the activation of PtdIns(3,4,5)P3-dependent kinases, such as PDK1, which, in turn, activates Akt [107]. Leptin is responsible for the stimulation of hypothalamic PI3K activity, which is mediated by IRS2. It also stimulates a signaling pathway that is similar to insulin in critical organs, such as the central nervous system (CNS), adipose tissue, the pancreas, and the liver [116]. This mechanism involves a PI3K-dependent activation of phosphodiesterase 3B (PDE3B) and a decrease in the levels of cAMP. Furthermore, the activity of PI3K is necessary for the sympathetic activation effects that are mediated by leptin. One of the most important components of leptin's signaling in the hypothalamus is the interaction between the PI3K/PDE3B/ cAMP pathway and the JAK2/STAT3 signaling cascade [117]. Leptin can elicit both activating and inhibitory reactions on PKC, which results in a reduction in insulin production from pancreatic islets in response to PKC stimulation and a decrease in Ca^2+^-dependent PKC activity inside the pancreas [118]. Additionally, leptin has the potential to block the component of the phospholipase C–PKC signaling pathway that is controlled by PKC and is involved in the process of insulin production [119].

## 16 THERAPEUTIC POTENTIAL OF LEPTIN REPLACEMENT TREATMENT

Leptin replacement therapy improves glycaemic control, insulin sensitivity, and lipid levels in individuals with lipodystrophy. Additionally, it reduces the size of the liver, decreases the accumulation of fat in the liver, and reduces the need for antidiabetic medication [120]. This medication has the ability to cure diabetes in animal models, regardless of their body weight or food consumption, regardless of whether it is type 1 or type 2 diabetes [121]. Individuals with lipodystrophy who have severe insulin resistance have positive effects from enhanced glycaemic control and increased insulin sensitivity. Leptin replacement therapy improves lipid profiles and decreases hepatic steatosis in individuals with congenital or acquired lipodystrophy [122].

Leptin also restores neuroendocrine and immunological functioning in individuals with congenital leptin deficiency and energy-deprivation illnesses, such as hypothalamic amenorrhea and anorexia. Leptin reduces the levels of amyloid beta and the phosphorylation of tau, hence enhancing cognitive performance in animal models of Alzheimer's disease [123]. This indicates that leptin has promise as a treatment. Leptin resistance often occurs in cases of obesity-related hyperleptinemia, which diminishes its effectiveness. Research aimed at enhancing leptin sensitivity has yielded limited results. Leptin replacement therapy may be beneficial in addressing metabolic and endocrine issues, particularly those resulting from a deficiency of leptin, such as lipodystrophy and congenital LDD [124]. Studies using CRISPR-Cas9, loxP, and leptin-neutralizing mouse models have shown that reducing leptin levels enhances metabolic sensitivity. The models consumed a reduced amount of food, had a lower increase in weight, and exhibited improved insulin sensitivity and glucose tolerance [125]. Endoplasmic reticulum (ER) stress in the hypothalamus leads to the development of leptin resistance. However, the application of pharmacological chaperones, such as 4-phenyl butyric acid and tauro-ursodeoxycholic acid, may enhance the sensitivity of leptin and improve its signaling pathways [126]. Melatonin, renowned for its ability to combat oxidative stress and inflammation, may also have therapeutic potential in addressing obesity associated with leptin resistance. This is achieved by specifically targeting lipid metabolism and endocrine signaling [127]. 1,3-Butanediol (BD) enhances hypothalamic ATP levels, hence decreasing endoplasmic reticulum (ER) stress and enhancing leptin sensitivity. This leads to a decrease in food consumption and body weight in mice with diet-induced obesity [128]. By focusing on phosphatases that have a negative impact on leptin signalling and by controlling hypothalamic inflammation, it may be possible to restore sensitivity to leptin. This emphasizes the intricate nature of leptin resistance and offers potential avenues for therapy [129]. Decreasing levels of leptin, alleviating endoplasmic reticulum stress, and using melatonin and 1,3-butanediol may enhance the sensitivity of leptin, offering potential therapy approaches for leptin resistance. By concurrently focusing on molecular pathways and reducing hypothalamic inflammation, these strategies have the potential to result in the effective treatments for obesity [130]. Limiting calories and other modifications to lifestyle may help patients lose weight and recover leptin sensitivity, which regulates hunger and protects against obesity-related diseases, including heart disease [131]. Leptin-based treatment helps obese people with leptin gene abnormalities by reinstating energy balance, glucose regulation, and neuroendocrine function. In most instances of eating-related being overweight, this medication is ineffective, especially when leptin levels are high. Research has combined leptin with sensitizing therapy to combat leptin resistance [132]. These medicines target leptin signaling pathway negative regulators SOCS3 and PTP1B. Research on mice suggests that these inhibitors may decrease weight growth, reduce food intake, and lower body weight. Changes in leptin receptor intracellular trafficking and endocytosis may help fight obesity [133]. Even with high leptin levels, most fat persons fail leptin treatment. Sensitizers may restore leptin sensitivity, boost weight loss, and increase externally given leptin's appetite-suppressing effects. Amylin and other sensitizers boost leptin effectiveness by increasing hypothalamic IL-6 and STAT3 pathway activity [134]. Celastrol boosts leptin sensitivity by inhibiting 6-phosphofructokinase in the muscle cells and activating AMPK [135]. Energy utilization adjustments reduce hunger and promote weight loss *via* changing glycolysis and fatty acid oxidation. The Japanese government authorized leptin-like metreleptin in 2013 [136]. It may decrease plasma glucose, boost insulin sensitivity, and lower the body's mass index and food consumption, according to research. Given these outcomes, it may help eliminate type 2 diabetes [137]. Low or high leptin levels are linked to cardiovascular disease and death. Leptin inhibits hunger in the hypothalamus and other regions of the brain when there is an abundance of energy [138].

## CONCLUSION

The findings of this study provide valuable insights into the pivotal role of leptin, a hormone that is essential for regulating energy balance, metabolism, and cardiovascular well-being. Leptin is essential for maintaining energy balance and controlling body weight *via* its influence on hunger, energy expenditure, and fat accumulation. Its main action occurs in the hypothalamus of the brain, where it triggers the JAK2/STAT3 signaling pathway. This pathway plays a crucial role in facilitating the effects of leptin on energy balance and glucose metabolism. However, individuals who are obese often have disruptions in this signaling mechanism, leading to the development of leptin resistance. Leptin resistance is a state in which the body, although having increased levels of circulating leptin, does not respond efficiently to the hormone. Obesity often results in this resistance, leading to a diminished ability to regulate food intake and energy expenditure. This exacerbates weight gain and leads to the onset of metabolic disorders, such as type 2 diabetes and cardiovascular disease. This research emphasizes the influence of leptin resistance on both metabolic functions and cardiovascular well-being. Higher concentrations of leptin are linked to elevated blood pressure, compromised arterial function, and an increased susceptibility to cardiovascular ailments, such as stroke and heart attack. Leptin resistance is a significant factor in the link between obesity and cardiovascular disease, as it exacerbates inflammation and oxidative stress, both of which contribute to these conditions. This study investigates the therapeutic effectiveness of leptin replacement therapy, especially in treating illnesses characterized by a deficiency of leptin, such as congenital lipodystrophy. Leptin treatment has shown promise in improving glycaemic control, lipid profiles, and general metabolic health. However, the effectiveness of the drug is significantly reduced in cases of leptin resistance, which is common in obese people. The study suggests that enhancing the sensitivity of leptin, a hormone responsible for controlling hunger and metabolism, may be accomplished *via* several approaches. These techniques include reducing the levels of leptin, focusing on certain metabolic pathways, and alleviating endoplasmic reticulum stress. By using these strategies, there is a possibility of creating new methods for treating various conditions. The finding underscores the need for continuous study to acquire a more profound comprehension of the processes behind leptin resistance and to develop efficacious therapies. It emphasizes the need to focus on medicines that may improve leptin sensitivity and successfully treat obesity and its associated metabolic and cardiovascular diseases. Acquiring a thorough comprehension of the complex interrelationships between leptin signaling and metabolic health can facilitate significant advancements in the management of obesity, diabetes, and cardiovascular disease. In summary, the research emphasizes the importance of leptin in maintaining metabolic and cardiovascular health, the challenges posed by leptin resistance, and the potential for therapeutic approaches targeting these pathways. It underscores the importance of targeting leptin resistance as a crucial factor in developing effective therapies for a range of health issues associated with obesity.

## Figures and Tables

**Fig. (1):**
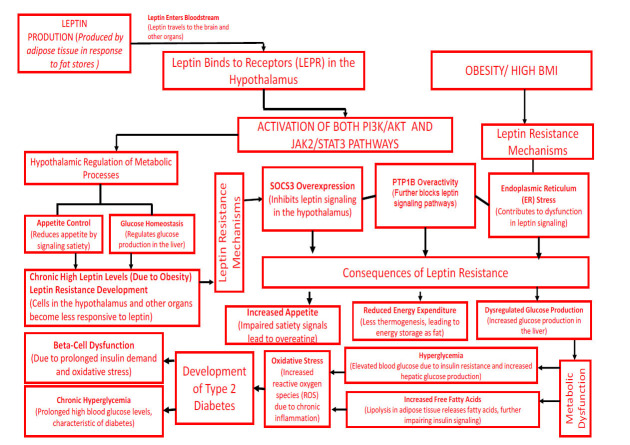
The role of leptin after binding to the hypothalamus and the impact of its resistance on the development of diabetes.

**Fig. (2):**
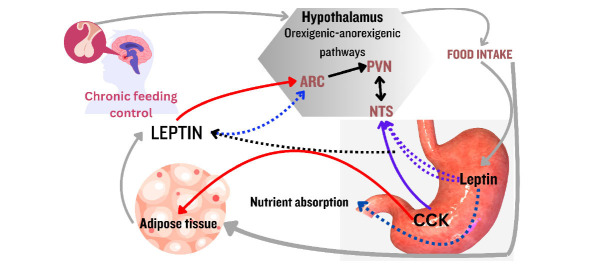
This diagram depicts leptin and cholecystokinin (CCK) from adipose tissue and the stomach regulating food intake and nutrient absorption through key hypothalamic regions, including the arcuate nucleus (ARC), paraventricular nucleus (PVN), and nucleus of the solitary tract (NTS).

**Fig. (3):**
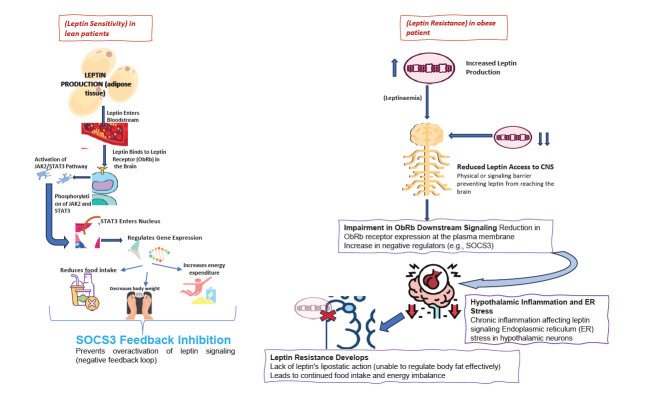
This figure shows leptin sensitivity in lean individuals, enabling energy balance, versus leptin resistance in obesity, where impaired signaling leads to weight gain and metabolic issues.

**Table 1:** Roles of various pathways and receptors in hypertension, CVD, and obesity.

**Receptors /Pathways**	**Role in Hypertension**	**Role in Cardiovascular Disease (CVD)**	**Role in Obesity**
Leptin Receptor (LEPR)	Genetic variations in LEPR linked to increased blood pressure and hypertension *via* sympathetic nervous system activation.	LEPR gene variations associated with increased risk of stroke, endothelial dysfunction, and arterial stiffness.	Leptin resistance in LEPR leads to impaired appetite control and increased body fat, exacerbating obesity.
JAK2/STAT3 Pathway	Dysregulation of this pathway leads to increased sympathetic activity, contributing to hypertension.	Impaired signaling in this pathway can increase the risk of vascular inflammation, contributing to CVD.	Critical in regulating energy balance; dysfunction contributes to leptin resistance and obesity.
SOCS3 (Suppressor of Cytokine Signaling 3)	Overexpression blocks JAK2/STAT3 signaling, worsening leptin resistance and contributing to hypertension.	Increased SOCS3 activity linked to chronic inflammation, leading to endothelial dysfunction and CVD risk.	SOCS3 inhibits leptin signaling, promoting leptin resistance and obesity, particularly in diet-induced obesity.
PTP1B (Protein Tyrosine Phosphatase 1B)	Inhibits leptin signaling pathways, exacerbating leptin resistance and potentially contributing to hypertension.	Impairment of leptin signaling by PTP1B may lead to increased cardiovascular risk through inflammation.	PTP1B inhibition may improve leptin sensitivity, offering potential therapeutic strategies for obesity.
PI3K (Phosphoinositide 3-Kinase)	Impacts insulin sensitivity and leptin signaling; disruptions can contribute to hypertension development.	PI3K signaling influences endothelial function, potentially contributing to vascular issues in CVD.	PI3K is involved in leptin and insulin signaling; disruption leads to increased fat storage and obesity.
SHP2 (Src Homology 2 Domain-Containing Protein Tyrosine Phosphatase)	Negative regulator of leptin signaling; its inhibition may help reduce hypertension by improving leptin sensitivity.	Associated with impaired vascular health and increased risk of atherosclerosis in CVD patients.	SHP2 affects leptin sensitivity and energy regulation, contributing to obesity when dysregulated.

## LIST OF ABBREVIATIONS

AgRP: Agouti-related Peptide
Akt: Protein Kinase B
AMPK: AMP-activated Protein Kinase
ARC: Arcuate Nucleus
BMI: Body Mass Index
C–PKC: Conventional Protein Kinase C
CCK: Cholecystokinin
CRISPR-Cas9: Clustered Regularly Interspaced Short Palindromic Repeats and CRISPR-associated protein 9
CVD: Cardiovascular Disease
ER: Endoplasmic Reticulum
FCH: Familial Combined Hyperlipidemia
FTO: Fat Mass and Obesity-associated Gene
GCKR: Glucokinase Regulatory Protein
IL-6: Interleukin-6
IRS-1: Insulin Receptor Substrate 1
JAK2: Janus Tyrosine Kinase 2
LDD: Lymphatic Dissemination of Disease
LEPR: Leptin Receptor
LncOb: Long Non-coding RNA Obesity-associated
MPTP: Mitochondrial Permeability Transition Pore
NO: Nitric Oxide
NTS: Nucleus of the Solitary Tract
ObRb: Obesity Receptor type B or Leptin Receptor B
PDE3B: Phosphodiesterase 3B
PDK1: 3-Phosphoinositide-Dependent Protein Kinase-1
PI3K: Phosphoinositide 3-Kinase
PI3K-PDE3B-cAMP: Phosphoinositide 3-Kinase-Phosphodiesterase 3B-Cyclic Adenosine Monophosphate Pathway
PKC: Protein Kinase C
POMC: Pro-opiomelanocortin
PTP1B: Protein Tyrosine Phosphatase 1B
PVN: Paraventricular Nucleus
RBP4: Retinol-binding Protein 4
SH2B1: Src-homology 2 Domain 1
SLC32A1: Solute Carrier Family 32 Member 1
SLE: Systemic Lupus Erythematosus
SOCS3: Suppressor of Cytokine Signaling 3
STAT3: Signal Transducer and Activator of Transcription 3
T2DM: Type 2 Diabetes Mellitus
TNF-α: Tumor Necrosis Factor-alpha
Trpm7: Transient Receptor Potential Melastatin 7
